# Preparation and identification of anti-breast cancer cells peptides released from yak milk casein

**DOI:** 10.3389/fnut.2022.997514

**Published:** 2022-08-26

**Authors:** Haofeng Gu, Lei Liang, Ziwei Zhu, Xueying Mao

**Affiliations:** ^1^School of Modern Agriculture and Biotechnology, Ankang University, Ankang, China; ^2^College of Food Science and Nutritional Engineering, Key Laboratory of Functional Dairy, Ministry of Education, China Agricultural University, Beijing, China

**Keywords:** hydrolysate, peptides, breast cancer cells, yak milk casein, identification

## Abstract

Yak milk casein (YMC) is the main protein in the yak milk. Peptides released from Yak milk casein (YMC) have multiple bioactivities, including anti-inflammation and immune-regulation, suggesting that these peptides might be able to inhibit cancer theoretically. However, the anti-cancer peptides from YMC have only been sparsely studied. Breast carcinoma is the most common carcinoma in women worldwide. Thus, the paper herein was to identify yak milk casein (YMC)-derived anti-breast cancer peptides *via* gel filtration, reversed phase high-performance liquid chromatography (RP-HPLC) and liquid chromatography electrospray ionization tandem mass spectrometry (LC-ESI MS/MS) for the first time. The inhibitory effects of the hydrolysates on the cell viabilities, cell cycles and apoptosis of breast cancer cells were evaluated with a cck8 kit and a flow cytometry. The result showed that YMC hydrolysates (YMCH) obtained by united hydrolyzation with trypsin (3 h) and alkaline protease (3 h) displayed the highest cell viability inhibition rate for MCF7 (20.74 ± 1.39%) and MDA-MB-231 (26.73 ± 2.87%) cells. Three peptides were identified in the RP-HPLC subfraction F3-4, and a nonapeptide (TPVVVPPFL) showed the most potent inhibitory effects on both cancer cells and displayed good gastrointestinal stability. TPVVVPPFL could induce G2-M cell cycle arrest in MCF7 cells and S cell arrest in MDA-MB-231 cells and induce apoptosis in both cancer cells. Moreover, *in silico* analysis indicated that the peptide had non-toxic and no inhibitory roles on P4502D6-enzyme. Together, this study shows that YMC is a good source of anti-breast cancer cells peptides.

## Introduction

Breast cancer is a refractory carcinoma among females, which is usually responsible for cancer-related death worldwide (1). Clinically, radiotherapy, chemotherapy, and surgical excision are the main strategies to fight against breast cancer; these treatments have improved survival rate and prognosis of patients, especially for the patients in the early stage (1). However, due to the cytotoxicity and genotoxicity of chemotherapeutics, anti-cancer drugs have various side effects such as alopecia, vomiting and lymphedema (2, 3). Thus, novel anti-cancer drugs with fewer side effects are encouraged to develop.

Peptides are characterized by low cytotoxicity, tumor-penetrating capability, and small length. These agents are promising and safe anti-cancer drugs (4). For example, Anginex, an artificial peptide targeting the anti-angiogenic proteins, is capable of inhibiting metastasis of osteosarcoma (5). Besides synthetic peptides, there are many anti-cancer peptides released from various foods such as rice bran (6), soybean (7), common bean (8), and bovine milk (9). Bovine milk is the richest source of anti-cancer peptides, and these peptides are primarily released from casein (α_*s*_, k, and β casein) (10), suggesting that casein is a good source of anti-cancer peptides. For example, peptides from buffalo and cow milk.

Cheddar cheeses inhibited colon cancer cells *via* inducing G0/G1 cell cycle arrest and apoptosis (11); a β casein derived peptide (PGPIPN) triggered the apoptosis in human ovarian cancer cells *via* targeting BCL2 signaling (9); INKKI, a peptides released from bovine β-Casein, inhibited tumor growth and cell viability *via* inducing apoptosis in Melanoma B16F10 cells (12). Like bovine milk casein, yak milk casein (YMC) is the main protein in Yak milk. Peptides released from YMC have multiple bioactivities, including anti-inflammation (0.5–1.0 mg/mL) (13), immune-regulation (0.10–0.50 mg/mL) (14) and anti-oxidation (2.5–5.0 mg/mL) (15). Considering that malignant tumor is characterized by high inflammation, high oxidative stress, and immune evasion, bioactive peptides derived from YMC might be able to fight against cancer in theory. However, there are no studies focusing on exploring the anti-cancer peptides derived from YMC.

Thus, the purpose of the paper herein was to prepare and identify the promising anti-breast cancer peptides from YMC and uncover the underlying mechanisms. This paper studied the YMC-derived anti-cancer peptides for the first time.

## Materials and methods

### Material and reagents

Yak milk casein (YMC) was provided by Treasure of Plateau Dairy Co., Ltd., China. Pepsin (142.6 U/mg), Dulbecco’s modified Eagle’s medium (DMEM), alcalase (278.8 U/mg protein), propidium iodide (PI), hydrocortisone and trypsin (206.2 U/mg protein) were obtained from Sigma-Aldrich (MO, United States). Cholera toxin (CT), CCK8 kit, cell cycle assay kit, and cell apoptosis kit, trypsin-EDTA solution and epidermal growth factor (EGF) were purchased from Beyotime (Shanghai, China). Horse serum (HS), antibiotic-antimycotic cocktail (AAC), and fetal bovine serum (FBS) was obtained from Giboc (NY, United States). MDA-MB-231 and MCF7 cells were kindly provided by the Lab of professor Zhengquan Yu at China Agriculture University.

### Preparation of yak milk casein hydrolysates

Yak milk casein (YMC) was dispersed in distilled water (5°g/100 mL) and successively hydrolyzed by trypsin (pH 8, 37^°^C, 3 h) and alcalase (pH 8.0, 55^°^C, 3 h). The amount of each enzyme used in the reaction was 1:20 (w/w, enzyme to substrate). YMCH at 0, 3, 3.5, 4, 5, and 6 h were gathered followed by inactivating proteinase activity (90^°^C, 15 min). All YMCH solutions were further gathered by centrifugation (2,500 × *g*, 15 min). The samples were freezing dried (−60*^o^*C, 0°pa, 48 h) with a lyophilizer (FD-1A-50, Qiao Yue, Shanghai, China) and stored at −20*^o^*C.

### Measurement of the hydrolysis degree

The hydrolysis degree of YMCH was measured following the previous method (16). Briefly, YMCH (0.5 mL) were diluted to 25 mL by sodium dodecyl sulfate (SDS) (1°g/100 mL), and phosphate belanced solution (PBS) (1 mL, pH 8.2) and 1 mL of trinitrobenzene sulfonic acid (0.1%, w/w) were added to 0.125 mL of the mixture. The mixed solution was incubated at 50°C for 1 h in the dark, and the reaction was terminated by 2 mL of HCl (0.1°M). After cooling for 30 min, the absorbancy of the resulting mixture was determined at 420 nm. L-leucine (0–5°mmol/L) was used as the standard solution. The standard curve was as follows: *Y* = 0.2283x+0.2212, *R*^2^ = 0.9959.

### Cell culture

The culture medium of MDA-MB-231 and MCF7 cells were DMEM containing 10% FBS and 1% AAC. The culture medium of MCF10A was DMEM/F12 containing CT (100°ng/mL), EGF (20°ng/mL), HS (5%), hydrocortisone (0.5°μg/mL), and insulin (10°ug/mL). All the cells were incubated using an incubator (MCO-20AIC, SANYO, Japan) with 5% CO_2_ and 95% humidity at 37°C.

### Determination of cell viability inhibition

Cell activity inhibition was conducted using a CCK8 kit according to the procedure offered by the manufacturer. The trypsinized cells were transferred into the 96-well plate (8,000 cells/well) and cultured for 24 h, then they were incubated with PBS (Control), YMCH (250°μg/mL) or synthetic peptides (0, 62.5, 125, 250, 500, and 1,000°μg/mL) for 48 h or 24–72 h. The CCK8 agent (20°μL) was pipetted into the well followed by incubation (37^°^C, 4 h). The absorbance of the medium was acquired at 450 nm using a microplate reader (Multiskan Spectrum, Thermo, Waltham, MA, United States). Finally, according to the below formulae, the cell viability and its inhibition rate were calculated.


$$Cellviability\left(\%\right)=\frac{\mathrm{Absorbance}\,\mathrm{of}\,\mathrm{medium}\,\mathrm{from}\,\mathrm{the}\,\mathrm{treatment}\,\mathrm{group}}{\mathrm{Absorbance}\,\mathrm{of}\,\mathrm{medium}\,\mathrm{from}\,\mathrm{the}\,\mathrm{control}\,\mathrm{group}}\times 100$$

$$Inhibitionrateofcellviability\left(\%\right)=\left(1\,\frac{\mathrm{Absorbance}\,\mathrm{of}\,\mathrm{medium}\,\mathrm{from}\,\mathrm{the}\,\mathrm{treatment}\,\mathrm{group}}{\mathrm{Absorbance}\,\mathrm{of}\,\mathrm{medium}\,\mathrm{from}\,\mathrm{the}\,\mathrm{control}\,\mathrm{group}}\right)\times 100$$

### Purification of yak milk casein hydrolysates by gel filtration

Yak milk casein hydrolysates, with the inhibitory effect on breast cancer cells, were purified using Sephadex G-25 (Cool Chemical Technology, Beijing, China) by the method outlined in our previous study (17). YMCH solution (20 mg/mL) was filtrated with a filter (0.45°μM) and purified using gel chromatography with Sephadex G-25 (16 mm × 80 cm). YMCH filtrate was eluted by deionized water (1.0 mL/min). Every 5 mL of the eluate was gathered. The absorbancy of each fraction at 220 nm was determined. The gathered samples were lyophilized and stored at −20°C.

### Purification of yak milk casein hydrolysates by reversed phase high-performance liquid chromatography

The fraction with the highest inhibition rate against the two cancer cells were isolated with semi-RP-HPLC following our previous method (17). Briefly, the sample solutions (2 mg/mL) was filtrated with a filter (0.45°μm) and separated by a semi-RP-HPLC equipped with a C18 column (250 × 4.6°mm, Shanghai Tong Micro Analysis Technology Co., Ltd., Shanghai, China). The elution program was as follows: Mobile phase A was 0.1% trifluoroacetic acid (v/v) solution; Mobile phase B was acetonitrile [10–90% (v/v)] in a linear gradient over a period of 40 min. The temperature and flow rate was 30°C and 0.5 mL/min, respectively. The elution was monitored at 220 nm. The subfractions were collected and lyophilized.

### Amino acid sequence assay

Amino acid sequence of the fractioned samples, with good anti-cancer efficiency, was analyzed by LC-ESI MS/MS as previously reported [Zhang et al. (18)]. Briefly, the fractioned samples were separated by the nanoAcquity nano HPLC system (Waters, MA), and the resulting sample was loaded to the MS system (Thermo Scientific, Waltham, MA, United States). The positive ion mode and full scan (100–2,200°m/z) were used in the MS system. AA sequences were identified following searching and alignment in the UniProt database.^1^

### Peptide synthesis

Peptides, the purities of which were > 95%, were fabricated using Fmoc solid-phase synthesis procedure and were carried out by Wu Xi Peptide Co., Ltd., China.

### Determination of cell cycle

Cell cycle determination was conducted using a cell cycle kit according to its instructions. Trypsinized cells (MDA-MB-231 or MCF7) were pipetted into the 6-well plate and cultured. When cells confluence reached 50%, peptides were added to the wells, and the treatment lasted 48 h. The cells were harvested and washed with PBS. After being fixed by cold 75% ethanol, cells were treated with RNase A followed by incubation with PI. Finally, the cell cycles of samples were measured by a BD flow cytometer (FACS101, Becton, Dickinson and Company, Franklin Lakes, NJ, United States).

### Determination of cell apoptosis

Cell apoptosis determination was conducted by Annexin V staining of externalized phosphatidylserine in apoptotic cells using a cell apoptosis kit. Then the proportion of the apoptotic cells was analyzed by a BD flow cytometry (FACS101, Becton, Dickinson and Company, Franklin Lakes, NJ, United States).

### Stability against simulated gastrointestinal digestion

Stability of peptides against simulated gastrointestinal digestion was performed according to the method outlined previously with slight modification (19). Briefly, peptide solution (5 mg/mL) was digested by pepsin (pH 1.2, 37°C) for 2 h. Then the pH of the resulting solution was altered to eight with NaOH (1.0°M) to inactivate pepsin, and the peptides solution was further digested by trypsin (2 h, 37°C). Finally, trypsin was inactivated by a hot water bath (95°C, 10 min). The ratio of enzyme to peptide was 1:100 (w/w) in the two digesting processes. The digested and undigested samples were loaded into HPLC (1260 Infinity, Agilent, Santa Clara, CA, United States), respectively, to evaluate the stability of the peptides. The ratio of enzyme to peptide was 1:100 (w/w) in the two digesting processes.

### Computer simulation evaluation

The solubility of peptides was assayed using an online tool^2^ (20). The inhibitory effects of peptides on human cytochrome P4502D6 enzyme were analyzed using BIOVA Discovery Studio (Beijing Tech-Box S&T Co., Ltd., Beijing, China). The toxicity of peptides was assayed by a Toxin Pred web server.^3^

### Statistical analysis

Results were expressed as the means ± SD. Statistical differences (*p* < 0.05) were identified by Duncan procedures in SPSS 20.0 (IBM Inc., Chicago, IL, United States). Different letters show statistical differences (*p* < 0.05).

## Results

### Anti-breast cancer cells effects and degree of hydrolysis of yak milk casein hydrolysates

**FIGURE 1:**
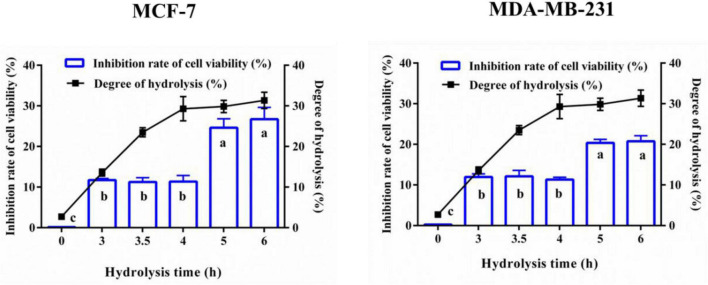
Anti-breast cancer cells activity and degree of hydrolysis of yak milk casein hydrolysates (YMCH). Yak milk casein (YMC) was united hydrolyzed by trypsin (3 h)+alkaline protease (0–3 h). MCF7 and MDA-MB-231 cells were incubated with the hydrolysates (final assay dose, 0.5 mg/mL) for 48 h, and the inhibitory effects of hydrolysates on the two breast cancer cells were evaluated by CCK8.

The cell viability inhibition rate of YMCH on breast cancer cells and hydrolyzing degree of YMCH were evaluated, as shown in Figure 1. YMCH showed a higher cell viability inhibitory rate on MCF7 than the intact YMC (*p* < 0.05). Moreover, compared with 3-h YMCH (11.94 ± 0.76% inhibition), 6-h YMCH with the highest hydrolyzing degree (31.36 ± 1.49%) displayed a more potent inhibitory effect on MCF7 cells (20.74 ± 1.39%) (*p* < 0.05). A similar result was found in the inhibition of MDA-MB-231 cells, while the inhibitory efficiency of YMCH against MDA-MB-231 was inferior to that of MCF7. These data suggested that hydrolyzing rate of YMCH was closely related to their inhibitory effects on mammary cancer cells, and MDA-MB-231 was more resistant to YMCH than MCF 7.

### Inhibiting effect of yak milk casein hydrolysates on breast cancer cells separated by gel chromatography and reversed phase high-performance liquid chromatography

**FIGURE 2:**
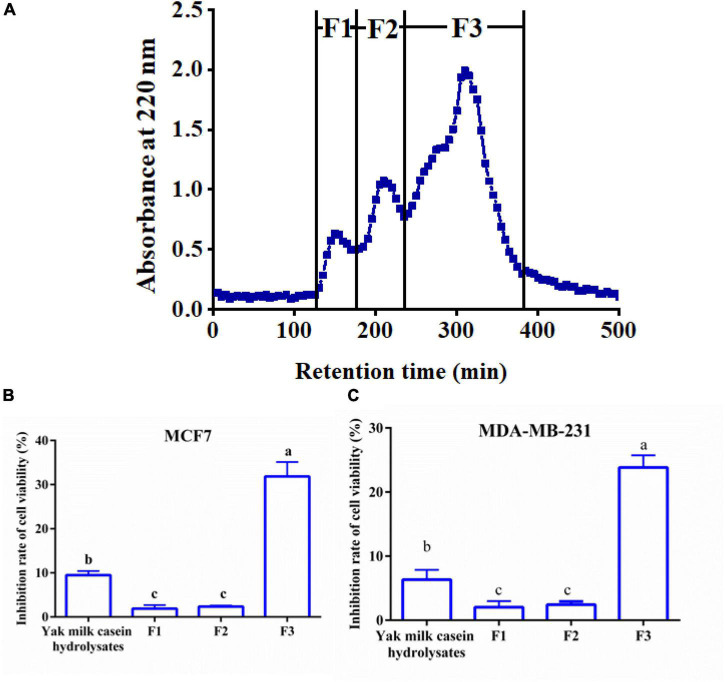
Elution profile **(A)** and anti-breast cancer cells activity **(B,C)** of yak milk casein hydrolysates (YMCH) fractions separated using a Sephadex G-25 gel column. The final dose of various YMCH was 0.1 mg/mL, the treatment time was 48 h.

**FIGURE 3:**
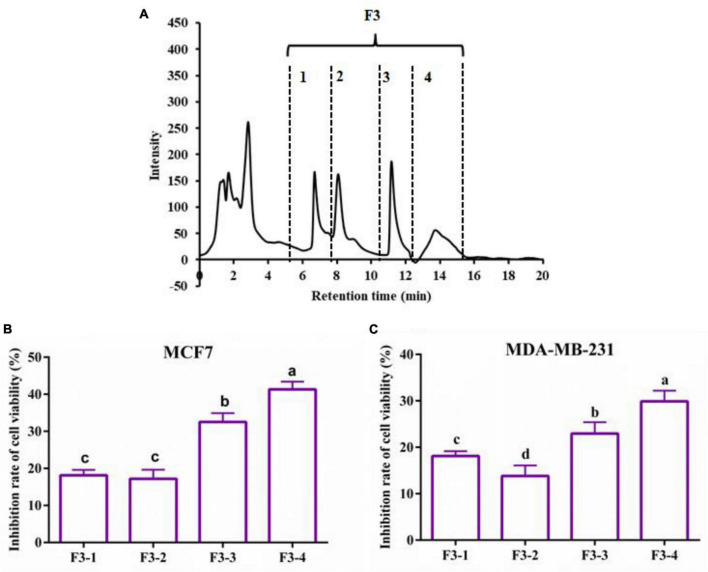
Reversed phase high-performance liquid chromatography chromatogram of F2 fraction **(A)** and the cell viability inhibition rate of subfractions against MCF7 cells **(B)** and MDA-MB-231 cells **(C)**. Both cancer cells were treated with 0.2 mg/mL of various subfractions (F3-1∼F3-4) for 48 h. The inhibitory effects of subfractions on the two breast cancer cells were evaluated by CCK8.

The 6-h YMCH with the highest anti-breast cancer cell activity was fractioned using G-25 gel filtration chromatography, and three fractions (F1-F3) were acquired (Figure 2A. Compared with the unpurified YMCH (9.47 ± 0.69%), resulting fraction F3 displayed the highest inhibition rate for MCF7 cell viability (31.83 ± 2.38%) (*p* < 0.05) (Figure 2B), a similar result was observed for the cell viability inhibition against MDA-MB-231 cells (Figure 2C). Further, fraction F3 were separated by RP-HPLC, and there were four subfractions in F3 including F3-1, F3-2, F3-3, and F3-4 (Figure 3A). Compared with other subfractions, F3-4 showed the highest inhibition rate against MCF7 (41.33 ± 2.08%) and MDA-MB-23 (29.87 ± 2.31) (*P* < 0.05) (Figures 3A–C).

### Identification of anti-breast cancer peptides

**FIGURE 4:**
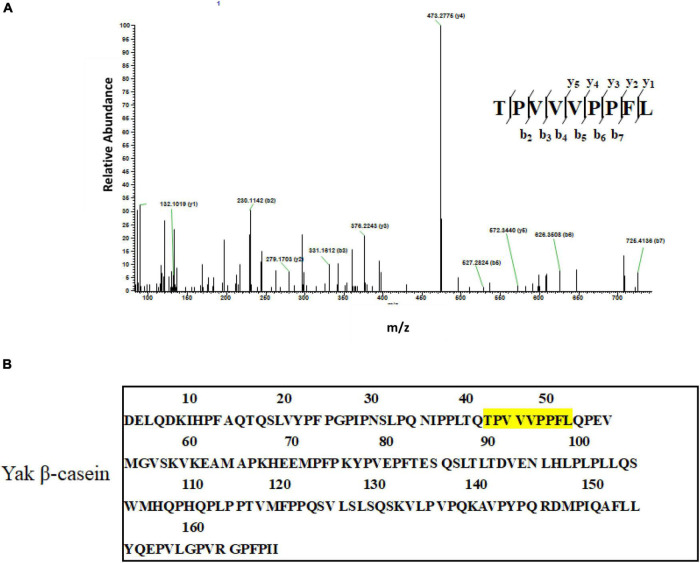
Identification of anti-breast cancer cells peptides. **(A)** LC-ESI MS/MS spectrum of the double-charged ion with m/z 510.2713. The spectrum fitted to TPVVVPPFL. **(B)** The primary structure of yak β-casein (UniProt KB number H2DZG3) and potential anti-cancer peptides identified in the yak casein hydrolysate fractions are colored yellow.

The AA sequences of subfraction F3-4 with the most potent anti-breast cancer cells activity were identified by LC-ESI-MS/MS. After interpreting the MS/MS sequence and searching and matching it in the online database of protein, the mass spectrum of the double-charged ion with m/z at 510.2713 matched to sequence TPVVVPPFL (Figure 4A), which was derived from β-casein (Figure 4B) (Supplementary Figure 1). Other two peptides, VAPFPEVFGK and NQFLPYPY, were identified, which were derived from α_*S1*_-casein and κ-casein, respectively (Supplementary Figure 2). Further, the GID stability and some drug-orientated properties were evaluated (Supplementary Figure 3). Supplementary Figure 3 showed that, compared with the two undigested peptides (TPVVVPPFL and NQFLPYPY), there were no new peaks in the RP-HPLC diagram of the two digested ones. The results suggested that the two peptides were stable in the simulated digestion condition. While some new peaks were found in the RP-HPLC diagram of the digested peptide (VAPFPEVFGK), showing that the peptide was hydrolyzed. Supplementary Table 1 showed that all peptides were poor water-soluble and no-toxic as well as no inhibition to human cytochrome P4502D6 enzyme.

### Inhibitory effects of synthetic peptides on breast cancer cells

**FIGURE 5:**
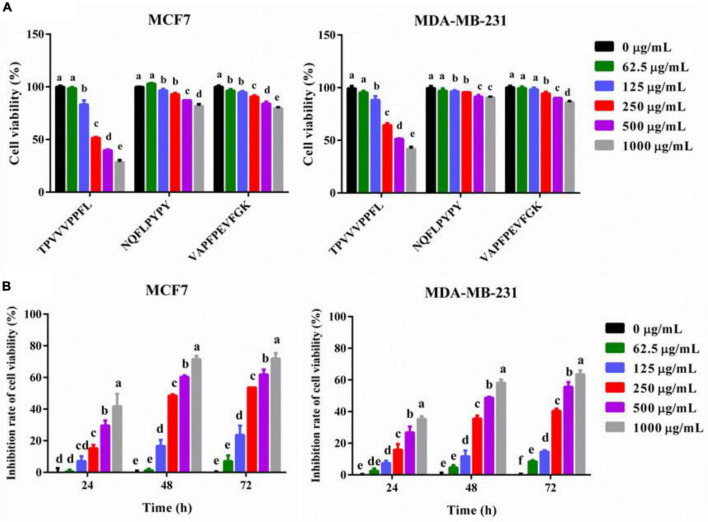
Synthesized peptides inhibited the cell viabilities of MCF7 and MDA-MB-231 cells. **(A)** The effect of synthesized peptides on the cell viabilities of the two cells. Both malignant cells were treated with synthesized peptides (TPVVVPPFL, NQFLPYPY, and VAPFPEVFGK) for 48 h at the concentration of 0, 62.5, 125, 250, 500, and 1,000°μg/mL. The cell viabilities were evaluated by CCK8. **(B)** The cell viability inhibition rate of TPVVVPPFL against MCF7 and MDA-MB-231 cells. The two malignant cells were incubated with TPVVVPPFL for 24–72 h at the concentration of 0, 62.5, 125, 250, 500, 1,000°μg/mL, respectively. The inhibitory effects of TPVVVPPFL on both breast cancer cells were evaluated by CCK8.

To further confirm the anti-breast cancer cells activity of synthetic peptides, cell viabilities of both breast cancer cells treated with different dosages of peptides were measured. All three synthetic peptides could inhibit the cells viability dose-dependently, and TPVVVPPFL showed the most potent anti-breast cancer effects compared with the other two peptides (*p* < 0.05) (Figure 5). The lowest cell activity of MCF7 and MDA-MB-231 cells incubated with TPVVVPPFL was 28.50 ± 1.50% and 41.80 ± 1.40%, respectively, so TPVVVPPFL was chosen for further studies. Figure 5B also showed that the cell viability inhibition rate of TPVVVPPFL was in a time dependent manner, and the IC_50_ of TPVVVPPFL was 250°μg/mL (MCF7) and 500°μg/mL (MDA-MB-231), respectively.

### TPVVVPPFL makes breast cancer cells arrest and apoptotic

**FIGURE 6:**
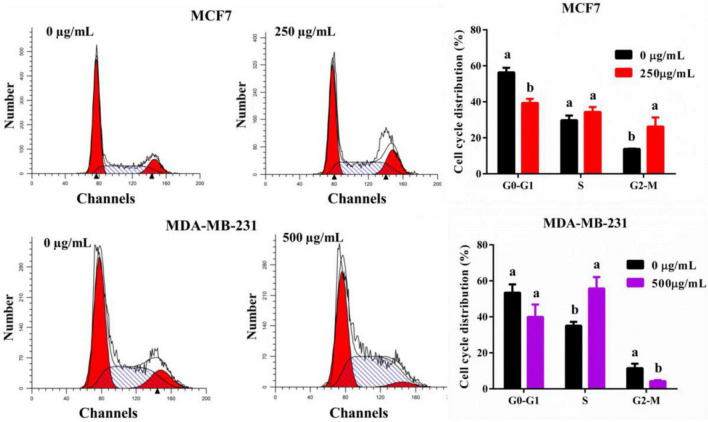
TPVVVPPFL elicited cell arrest of mammary carcinoma cells. MCF7 cells were incubated with TPVVVPPFL for 48 h at the concentration of 250°μg/mL. MDA-MB-231 cells were incubated with TPVVVPPFL for 48 h at the concentration of 500°μg/mL. The cell cycle distributions were analyzed using flow cytometry after treatment with PI.

**FIGURE 7:**
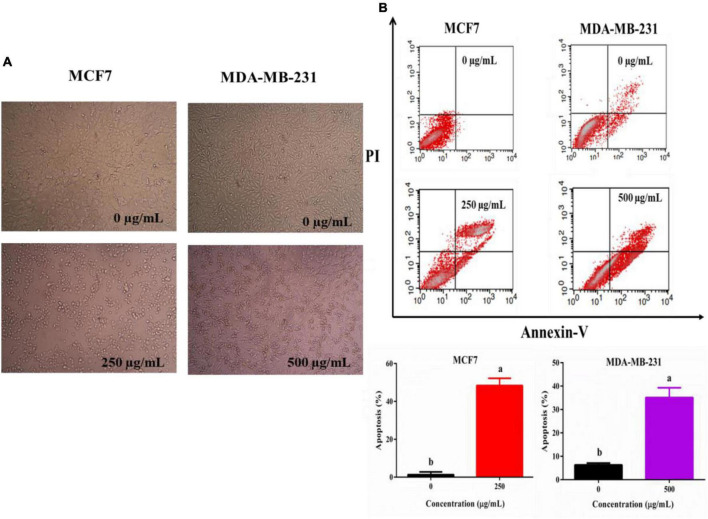
TPVVVPPFL induced morphology alteration **(A)** and apoptosis of mammary carcinoma cells **(B)**. MCF7 and MDA-MB-231 cells were incubated with TPVVVPPFL for 48 h at the concentration of 250 and 500°μg/mL, respectively. The image of the two cells was acquired by the inverted microscope, and the apoptotic cells were measured using a flow cytometry after treatment with Annexin-V and PI.

To analysis the reason for the anti-cancer effect of TPVVVPPFL, cycle arrest and apoptosis of both carcinoma cells were evaluated (Figure 6). TPVVVPPFL increased the G2/M amount of MCF7 cells and the S amount of MDA-MB-231 cells dramatically (*p* < 0.05), suggesting that TPVVVPPFL induced the G2/M arrest for MCF7 and S arrest for MDA-MB-231. Furthermore, TPVVVPPFL caused the noticeable morphologic changes (shrinking to round in shape) in both breast cancer cells (Figure 7A), which was related to the cells apoptosis. Expectedly, TPVVVPPFL treatment made MCF7 and MDA-MB-231 apoptotic. The apoptosis rate was 48.37 ± 2.74% and 35.11 ± 2.95%, respectively, (Figure 7B).

## Discussion

Breast carcinoma is the most common carcinoma in women worldwide (1). Although various efforts have been made to treat cancer, the side effects of chemotherapeutics due to the genotoxicity impair their anti-cancer efficiency (1). Thus it is needed to develop more natural and safer drugs. Recently, food-derived anti-cancer peptides have gained extensive attentions, and peptides released from YMC have many bio-activities (anti-oxidation, immune regulation, anti-inflammation) (13, 14), suggesting that YMC might be a good source of anti-cancer peptides. In the present study, 6-h YMCH (0.5 mg/mL) prepared by united hydrolyzation [trypsin (3 h)+ alcalase (3 h)] displayed anti-breast cancer cells effect (cell viability inhibition rate for MCF7 cells, 20.74 ± 1.39%). The anti-cancer effect of YMCH herein was inferior to the protein hydrolysates (1 mg/mL) from tuna dark muscle (30% or more) (21). These differences might be due to the variation of protein, concentration and enzyme used in the studies. This study is the first time to report the anti-cancer effect of YMCH. The anti-cancer effects of YMCH depended on the hydrolysis degree. A higher hydrolysis degree is commonly related to the lower molecular weight of the hydrolysates (17). For example, germinated soybean proteins hydrolysates (5–10°kDa) displayed a higher anti-proliferation effect on human colon cancer cells than hydrolysates with higher molecular weight (> 10°kDa) (7). A similar result was reported for the anti-cancer effect of hydrolysates released from defatted rice bran protein (6). These reports are in line with our results. While other researchers also showed that the degree of hydrolysis was not related to the anti-cancer effect of protein hydrolysates (21). Nevertheless, the degree of hydrolysis is one of the factors for the anti-cancer effect of protein hydrolysates, and the relationship between them still needs further investigation.

Other factors are also closely related to the anti-cancer effects of peptides, including the original proteins, composition and sequence of AA and hydrophobicity. Most of the milk-derived anti-cancer peptides are released from bovine casein, especially from β-casein. For example, PGPIPN (3 × 10^–3^−3 × 10^–2^ mg/mL) (9), INKKI (1.7°μM) (12) and LLYQEPVLEGPVRGPFPIIV (10°μg/mL) (22) are all released from β-casein and have an inhibitory effect on ovarian cancer cells, melanoma cells, and breast cancer cells, respectively. In the present study, anti-breast cancer peptides were found in YMC, which was homologous with bovine casein, and the most potent anti-cancer peptide (TPVVVPPFL) also derived from β-casein (f 38-46). Additionally, the occurrence frequency of some AA in casein-derived anti-cancer peptides is commonly higher than other AA, such as P, L, Q, V, K, F, and H (10). Herein, three identified anti-cancer peptides (TPVVVPPFL, VAPFPEVFGK, and NQFLPYPY) were mainly composed of AA (P, V, F, L, K, and Q) commonly found in casein-derived anti-cancer peptides. Meanwhile, the hydrophobicity is positively related to the anti-cancer effects, and hydrophobic amino acids in the alpha-helix of peptides are in favor of penetrating cell membrane, which can enhance the anti-cancer effects (23). Generally, the fraction coming out lastly in the RP-HPLC profile is usually more nonpolar than other fractions, so F1-4 subfraction is more nonpolar and hydrophobic. This is why most of the AA in the three peptides identified in F1-4 subfraction was hydrophobic, and the three peptides displayed poor solubility. The notable hydrophobicity of F1-4 subfraction and the three peptides were partly contributed to their anti-cancer effects.

Some properties such as toxin, human cytochrome P4502D6 enzyme inhibition and stability are vital factors determining the practical application of bioactive peptides (17). Human cytochrome P450 enzyme is the principal metabolic enzyme for biotransformation and metabolism of xenobiotics (such as drugs) (24). The inhibition of the enzyme is positively related to the metabolism and excretion of drugs (24). In the present study, three identified peptides displayed non-toxic, which might because they all derived from dietary YMC. Moreover, all peptides had no inhibitory effects on the enzyme, suggesting that all peptides enjoy available drug-oriented properties and deserve further study. In addition, because most drugs are taken orally, gastrointestinal stability is essential for their pharmacologic effect (25). In the present study, TPVVVPPFL and NQFLPYPY displayed good stability against GDI, suggesting the availability of oral administration for the anti-cancer peptide. Generally, trypsin is responsible for the digestion of the most of the ingested proteins; the identified peptides herein were released by the trypsin and alkaline protease, thus they were resistant to the GD.

Finally, inducing cell cycle arrest is a common mechanism underlying the anti-cancer effects of most chemotherapeutics (26). The cell cycle, a vital biological procedure, is composed of G0, G1, S, G2, and M phases (27). In the present study, the identified peptide (TPVVVPPFL) induced G2/M arrest in MCF7 cells and S arrest in MDA-MB-231 cells, which were partly responsible for the inhibitory effects of TPVVVPPFL on both breast cancer cells. Similar anti-cancer effects were also found by others. For example, peptides from the extract of Cheddar cheese induced the G2/M arrest in H-1299 cells (28); peptides derived from water buffalo cheese whey induced G1/G0 arrest in colon cancer cells (CaCo2) (29). Theoretically, once the cell cycle arrest happens, apoptosis might be the final fate of cells (30). Expectedly, TPVVVPPFL made both mammary cancer cells apoptotic. Moreover, MDA-MB-231 was more resistant to the peptide, and the concentration of peptides used in MDA-MB-231 was higher than MCF7 cells (500°μg/mL *vs.* 250°μg/mL), and a similar trend was found in the inhibition of cell viability. This discrimination might be due to the multi-drug resistance of the cell, a triple-negative breast carcinoma cell line which is more refractory and aggressive than other types of breast cancer cells (31).

## Conclusion

A novel anti-breast cancer cell peptide (TPVVVPPFL) was identified from YMCH, and the peptide inhibited breast carcinoma cells partly *via* making cell arrest and apoptotic. In all, the present study first indicated that the peptide derived from YMC was promising in the inhibiting breast carcinoma.

## Data availability statement

The original contributions presented in this study are included in the article/Supplementary material, further inquiries can be directed to the corresponding authors.

## Author contributions

All authors contributed to the methodology, validation, supervision, funding acquisition, and approved the submitted version.

## Footnotes

1
https://www.uniprot.org/

2
https://pepcalc.com/

3
http://crdd.osdd.net/raghava/toxinpred/index.html
