# Supplementary material for: Preparation and identification of anti-breast cancer cells peptides released from yak milk casein
Source: Front Nutr. 2022 Aug 26;9:997514. doi: 10.3389/fnut.2022.997514 (PMC9462664; doi:10.3389/fnut.2022.997514)
Supplement: Supplementary file 1 [file Data_Sheet_1.docx]

**Preparation and identification of anti-breast cancer cells peptides released from** **yak milk casein**

Hao-feng Gu^1,2^, Lei Liang^1^, Ziwei Zhu ^2^ and Xue-ying Mao^2^

^1^School of Modern Agriculture & Biotechnology, AnKang University, Ankang 725000, China;

^2^College of Food Science and Nutritional Engineering, Key Laboratory of Functional Dairy, Ministry of Education, China Agricultural University, Beijing 100083, China.

Correspondence to Xueying Mao and Hao-feng Gu.

Xue-ying Mao, [maoxueying@cau.edu.cn](mailto:maoxueying@cau.edu.cn).

Hao-feng Gu, ghf_ghf@163.com

**Supplementary data**

Supplementary figure 1


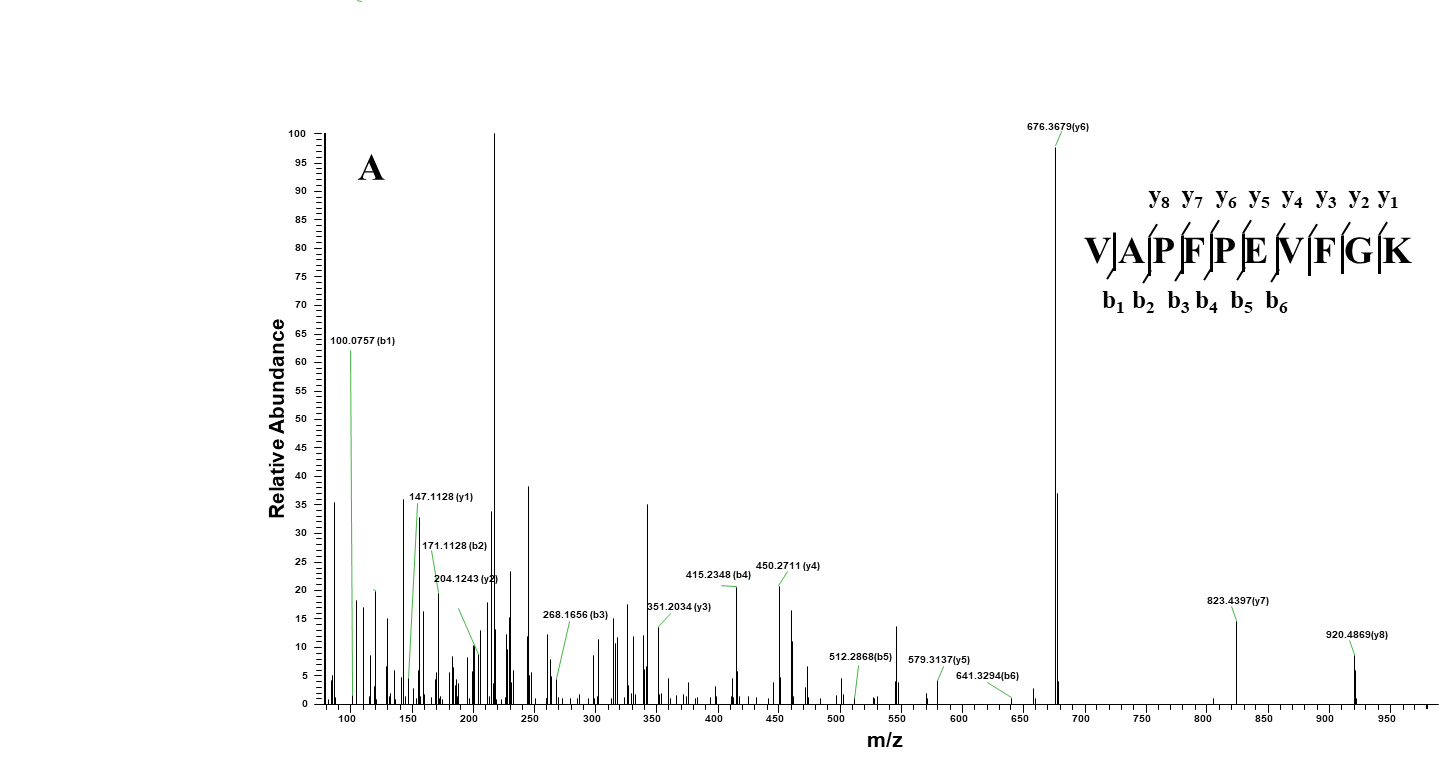


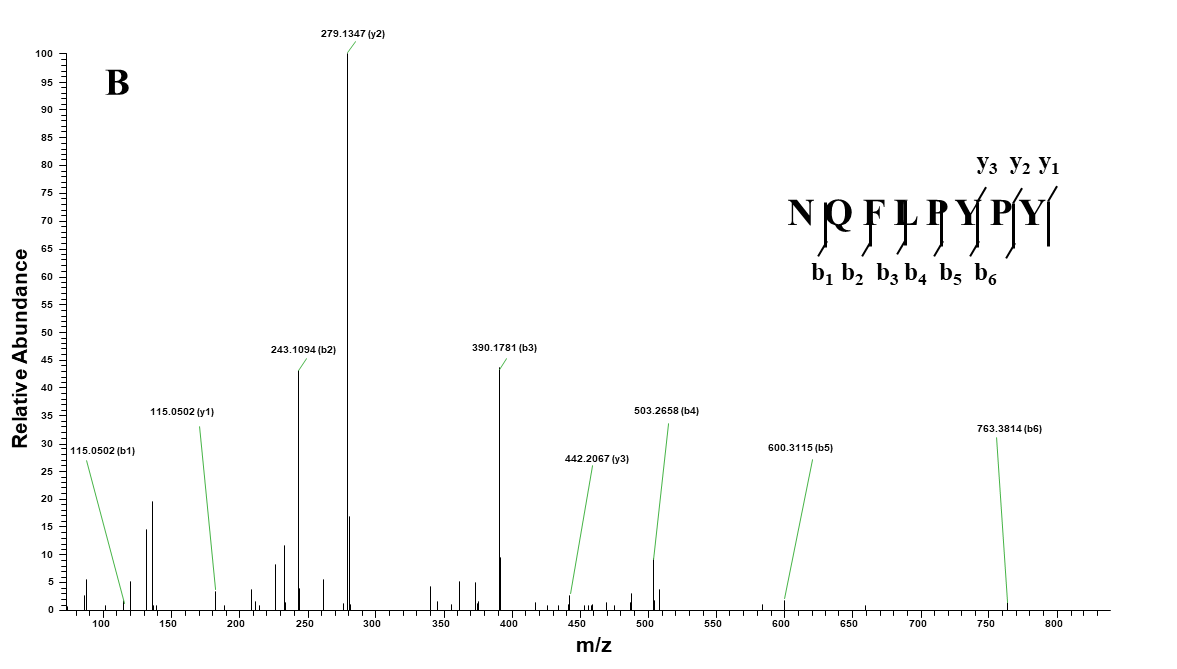


Figure S1 LC-ESI MS/MS spectrum of double-charged ion with m/z 545.8015 (A) and 521.2574 (B). The MS/MS spectrum was matched to peptide VAPFPEVFGK (A) and NQFLPYPY (B), respectively.

Supplementary figure 2


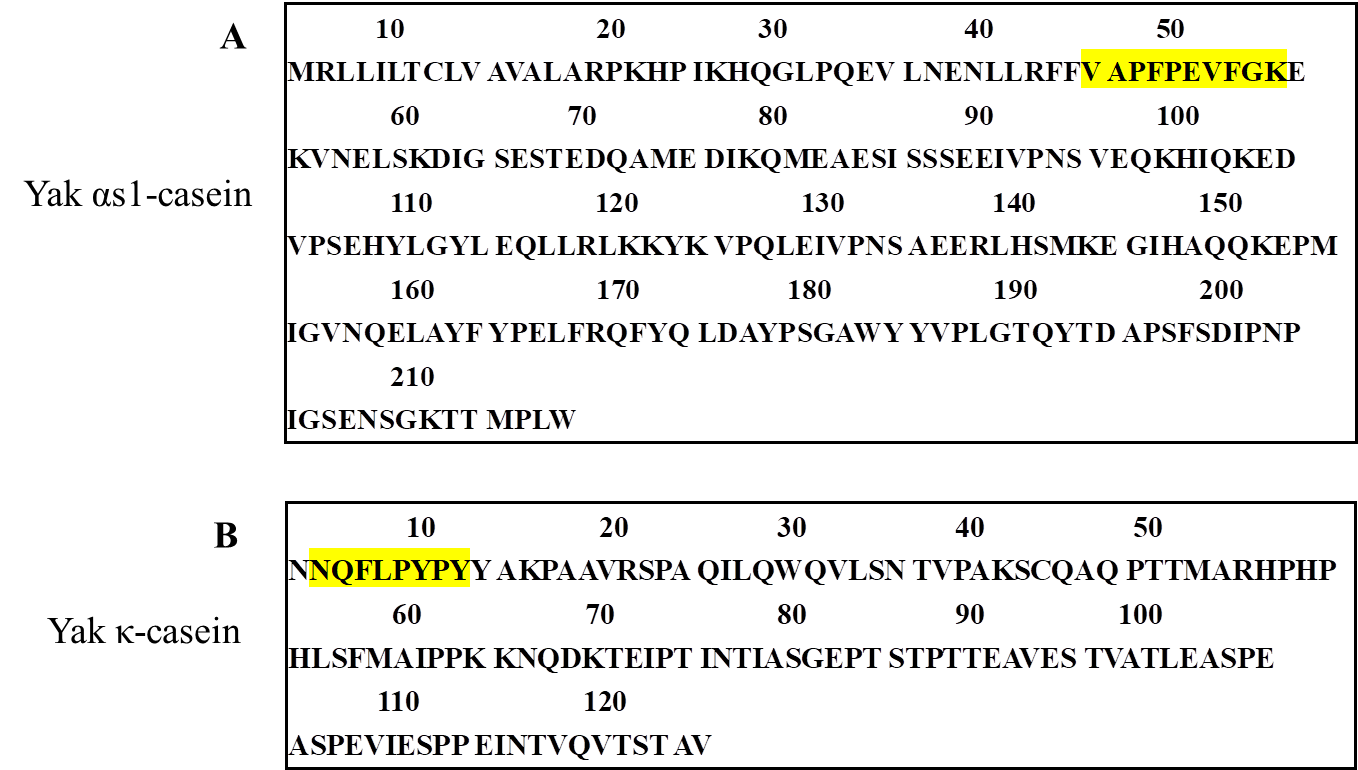


Figure S2 The primary structure of yak αs1-casein (UniProt KB number A0A344X7B7) (A), yak κ-casein (UniProt KB number O19178) (B), and potential anti-cancer peptides identified in the yak casein hydrolysate fractions are colored yellow.

Supplementary figure 3


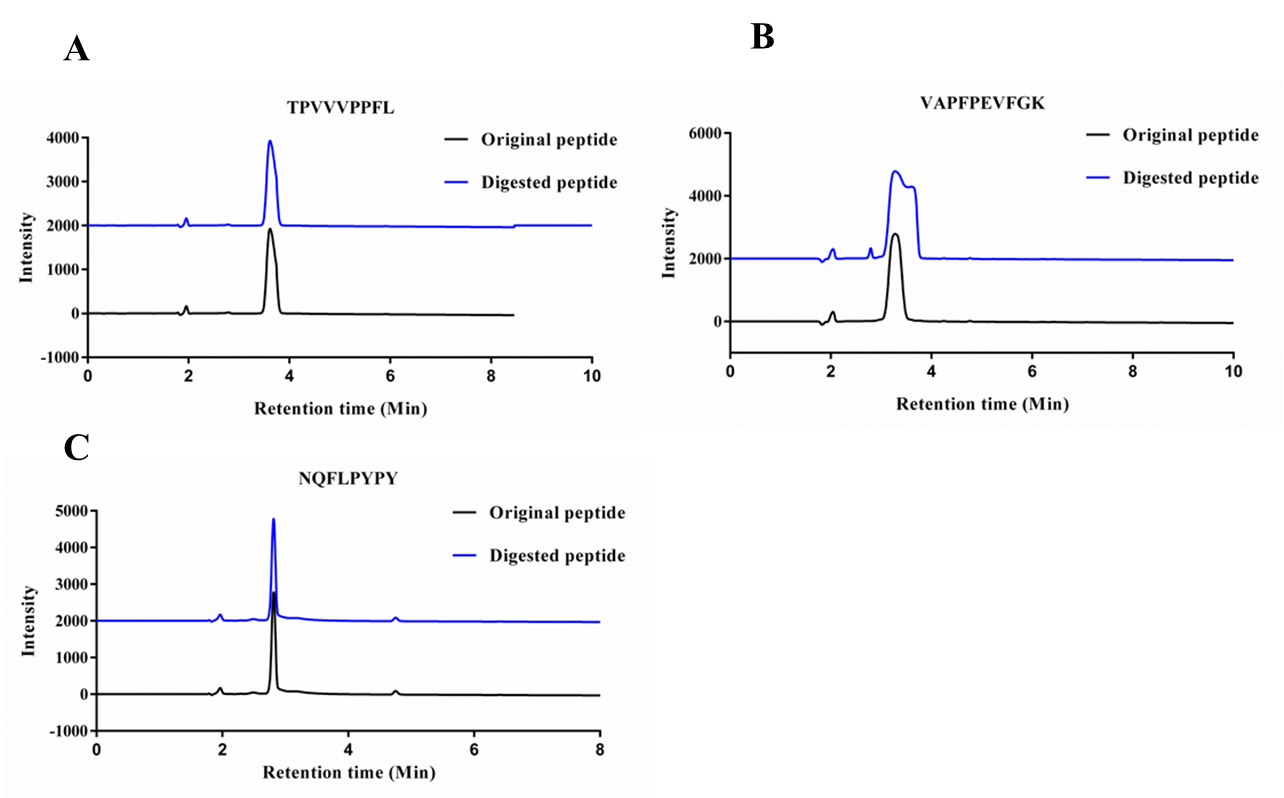


Figure S3 RP-HPLC chromatogram of (A) TPVVVPPFL, (B) VAPFPEVFGK and (C) NQFLPYPY before and after simulated gastrointestinal digestion.

Supplementary table 1

Table S1 The origin and predicted physicochemical properties of identified peptides in F3 fraction acquired by Sephadex G-25 gel filtration chromatography.

| **Peptide** | **Origin** | **Water-stability** | **Toxicity** | **Human cytochrome P4502D6 enzyme inhibition** |
| --- | --- | --- | --- | --- |
| TPVVVPPFL | yak β-casein f (38-46) | poor | Non-Toxin | Non inhibition |
| VAPFPEVFGK | yak αs1-casein f (40-49) | poor | Non-Toxin | Non inhibition |
| NQFLPYPY | yak κ-casein f (2-9) | poor | Non-Toxin | Non inhibition |
